# Biomechanical effects of sensorimotor orthoses in adults with Charcot-Marie-Tooth disease

**DOI:** 10.1186/1757-1146-8-S2-O39

**Published:** 2015-09-22

**Authors:** Caleb Wegener, Katrin Wegener, Richard Smith, Karl-Heinz Schott, Joshua Burns

**Affiliations:** 1 Sydney Arthritis and Musculoskeletal Research Network, The University of Sydney, New South Wales, Australia; 2 Shoe Tech Pty Ltd, Pedorthic Clinic, Dee Why, New South Wales, Australia; 3 Paediatric Gait Analysis Service of New South Wales, The Children's Hospital at Westmead, Sydney, New South Wales, Australia

**Keywords:** Charcot-Marie-Tooth disease, inherited neuropathy, foot orthoses, footwear

## Background

Charcot-Marie-Tooth disease (CMT) is an inherited neuropathy causing progressive weakness, foot deformity and difficulty walking. Clinical anecdotes suggest orthoses designed on the ‘sensorimotor’ paradigm are beneficial for improving gait in CMT.

## Methods

Eight males and two females with CMT aged 31-68 years were fitted with pedorthic shoes and custom-made sensorimotor orthoses were randomly tested at baseline and after a 4-week adaptation period. 3D multi-segment foot kinematics was collected with detachable markers through windows in the shoe. In-shoe plantar pressures were collected as well as EMG, lower limb kinematics, kinetics and self-reported comfort, stability, cushioning and footwear condition preference.

## Results

Compared to the shoe only condition, sensorimotor orthoses increased midfoot eversion and plantarflexion, increased ankle eversion and produced small but significant changes at the knee and hip indicating increased internal rotation. The orthoses increased medial ground reaction forces and increased pressure at the heel, midfoot and toes. There were minimal effects on EMG. The sensorimotor orthoses were rated higher for comfort, cushioning, stability and preference.

## Conclusions

Sensorimotor orthoses produce changes in kinematic, kinetic and pressure variables in adults with CMT and are regarded as more comfortable, cushioned and stable during walking. The walking ability of patients with CMT might improve with foot orthoses designed according to the sensorimotor paradigm. However, a larger randomised controlled trial is necessary to evaluate the long term self-reported benefits of sensorimotor orthoses.

